# Pre- and during-labour predictors of low birth satisfaction among Iranian women: a prospective analytical study

**DOI:** 10.1186/s12884-020-03105-5

**Published:** 2020-07-14

**Authors:** Jila Nahaee, Sakineh Mohammad-Alizadeh-Charandabi, Fatemeh Abbas-Alizadeh, Colin R. Martin, Caroline J. Hollins Martin, Mojgan Mirghafourvand, Hadi Hassankhani

**Affiliations:** 1 Students’ Research Committee, Faculty of Nursing and Midwifery, Tabriz University of Medical Sciences, Tabriz, Iran; 2 Social Determinants of Health Research Center, Department of Midwifery, Faculty of Nursing and Midwifery, Tabriz University of Medical Sciences, Tabriz, Iran; 3 Women’s Reproductive Health Research CenterTabriz University of Medical Sciences, Tabriz, Iran; 4 Institute for Clinical and Applied Health Research (ICAHR), University of Hull, Hull, UK; 5 School of Health, Edinburgh Napier University, Edinburgh, UK; 6 Department of Midwifery, Faculty of Nursing and Midwifery, Tabriz University of Medical Sciences, Tabriz, Iran; 7 Department of Medical and Surgical Nursing, Faculty of Nursing and Midwifery, Tabriz University of Medical Sciences, Tabriz, Iran

**Keywords:** Satisfaction, Risk factors, Predictors, Childbirth, Labour, Iran, Ethical code: IR.TBZMED.REC.1397.624.

## Abstract

**Background:**

Maternal childbirth dissatisfaction has short- and long-term negative effects on the mothers’ health and life, as well as on relation with her child and family. Due to lack of studies in Iran and other counties, we aimed to determine pre- and during- labour predictors of low birth satisfaction.

**Methods:**

Seven hundred women with low risk singleton pregnancy participated in this prospective analytical study. The participants were hospitalized for vaginal delivery with fetus in cephalic presentation and gestational age of 37^0^–41^6^ at two teaching centers in Tabriz (Iran). Woman characteristics, anxiety state (using Spielberger inventory) and dehydration were assessed at cervical dilatation of 4–6 cm. Iranian (Persian) birth satisfaction scale-revised was applied 12–24 h after birth. Multiple linear regression was used to determine the predictors.

**Results:**

Excluding 26 women who were outliers, 674 women were analyzed. The mean birth satisfaction score was 23.8 (SD 6.5) from an attainable score of 0–40. The during-labour predictors of low birth satisfaction score were severe and moderate anxiety, labour dystocia, insufficient support by staff, vaginal birth with episiotomy and tear, emergency cesarean section, labour induction and labour augmentation with oxytocin, and woman dehydration. The pre-labour predictors included being primiparous, sexual and emotional violence during pregnancy, gestational age of 40^0^–41^6^, preference for cesarean section, no attendance at pregnancy classes, and insufficient household income. The proportion of the variance explained by the during-labour variables was 75%, by pre-labour variables was 14% and by overall was 76%.

**Conclusions:**

The controllable during-labour predictors explains most of the variance of the satisfaction score. It seems that responding to women’s physical and psychological needs during labour and applying less interventions could improve women’s childbirth satisfaction.

## Background

High quality healthcare can ensure social justice and preserve client dignity [1]. The multidimensional aspects of healthcare provision are receiving increasing attention within the framework of healthcare quality determined by the World Health Organization (WHO) [2]. Nowadays, patient satisfaction is one of the major outcomes in healthcare and its assessment is considered a very important indicator of healthcare quality [3, 4].

According to WHO guidelines, not only should childbirth be safe, but it should also provide a pleasant experience for all women regardless of their socioeconomic condition. Care during labour and childbirth should be woman-centred and be provided for women and newborns based on a comprehensive approach that is evidence-based and in line with human rights [5].

Through creating a negative experience of childbirth, dissatisfaction with childbirth can induce many problems such as woman psychological disorders, disrupted parent-child relations, severe fear of childbirth, sexual dysfunction [6], disrupted husband-wife relations, post-traumatic stress disorder [7], and reduced inclination to have another child or long intervals between pregnancies [8].

The reported proportion of childbirth satisfaction are varied in different countries; 82% in Australia [9], 52% in South Africa [10], 88% in Ethiopia [11], 21% in Eritrea [12] and 63% in Iran [13]. Difference in the assessment method may have affected the results. However, childbirth satisfaction is a complex and multidimensional construct influenced by various factors. Therefore, the variation can be attributed to the differences in women’s expectations and quality of care, including the caregiver’s emotional support for women, the patient-physician relationship, encouragement received by women from the midwife or the physician and active involvement of women in decision-making about childbirth [14–16].

In some studies, a high level of childbirth satisfaction was also associated with low educational attainment [10, 11], positive attitude to childbirth [17], planned pregnancy, women’s knowledge of the stages of labour, lower labour pain intensity [18], administration of analgesics during labour [19], short labour [9], and childbirth by cesarean section [20]. However, the studies are limited and there are contradictory results about the associated factors. For example, in a study conducted in Italy, the results showed that women with a higher level of educational attainment undergoing vaginal birth were more satisfied with childbirth compared to less educated ones [21]; or a study conducted in Iran indicated that there was no correlation between mode of childbirth and level of satisfaction [22]. Also, in most of the previous studies, only a few number of possible risk factors have been investigated.

The most effective and least costly way of providing better and more suitable services is to find factors that influence patient dissatisfaction and attempt to eliminate them [4]. Therefore, considering the lack of sufficient evidence, we aimed to determine the risk- and predictive- factors of low childbirth satisfaction using an integrated and collaborative pre- and during- labour factors to help formulate more effective intervention strategies to improve women’s satisfaction with childbirth.

## Methods

This paper was extracted from a more extensive prospective case-control study entitled “birth satisfaction and predictors of prolonged labor” in which women with labour dystocia (cases) were compared with those with no dystocia (controls). Most of possible risk factors were assessed before identification of the dystocia.

In this prospective analytical study, data about most of possible predictors was collected during labour and data about childbirth satisfaction was gathered 12–24 h postpartum. Participants were selected from two hospitals (Taleghani and Al-Zahra), the only teaching maternal hospitals in Tabriz. With a population of 1.8 million, it is the sixth most populous city in Iran and is the capital city of East Azerbaijan province [23]. Taleghani is a tertiary hospital for referral from other centres in the province and Al-Zahra is a tertiary specialised gynaecology and obstetrics hospital for referral from other centres in the province, and also from nearby provinces with a total population of about 10 million [23]. The childbirth rate in each of these hospitals is about 500 births per month. In Iran, the majority (96.7%) of births occur in hospitals [24].

In these hospitals, an intravenous cannula was inserted on admission of women to the delivery room, with Ringer’s solution infusion commenced only upon physician orders, usually for labour induction or augmentation of labour. Remifentanil in low dose (0.01 to 0.03 μg/kg/min) was administered in case of normal foetus cardiogram and a minimum cervical dilatation of 5 cm, after obtaining the informed written consent from participants. The foetal heart and uterine contractions were regularly monitored with cardiotocogram devices and participants could mobilise out of bed for short periods of time. The participants had access to food and/or drink (based on stage of labour); however, since participants were lying on the bed and could not have a companion, and there was not enough staff, they were usually not given adequate liquids. In every shift, one midwife was responsible for caring two to three participants, and to observe foetal heart and uterine contraction. The obstetrics residents had direct responsibility for examinations and prescriptions during labour and delivery. The vaginal births were carried out by obstetrics residents, interns, midwifery students, or midwives.

Inclusion criteria were healthy women with a singleton uncomplicated pregnancy who were hospitalized for vaginal delivery with fetus in cephalic presentation and gestational age of 370–416 and had history of less than three births. Women with planned cesarean section (C-section), history of C-section at previous pregnancies, pre-labor contraindications for vaginal birth, and any abnormality in bone or soft tissues in pelvic cavity were excluded. Only those with emergency C-sections who were at least 3 h hospitalized in the labor room were included.

Participants were recruited at the labour wards, by completing the eligibility checklist through interview and vaginal examination (to determine cervical dilation, foetal presentation, and abnormality of pelvic). Written informed consent was obtained from all participants. To assure that women are definitely informed about content of the consent form, at first the investigator (PhD student, a highly experienced midwife) briefly explained contents of the form to each participant. Then, she gave them enough time to study the form and consult with family members through phone if needed. The investigator asked participants about the main parts of the form to ensure their understanding before they signed the consent form. Also, at time of collection of data about birth satisfaction, i.e.12–24 h after birth, the investigator got oral informed consent from all participants. If the woman age was under 16 years, written informed consent was also obtained from their husbands before data collection.

The socio-demographic questionnaire (Appendix 1), anxiety state inventory and dehydration checklist were completed at cervical dilatation of 4–6 cm using the medical records, interview with the women, or examination. The interviews were done between uterine contractions when the woman was relax.

The data extracted from the records were age, parity and gestational age. The other socio-demographic were collected through interview with the women including: education, employment, perceived sufficiency of household income, intention to pregnancy, attendance at birth preparation classes, smoking, alcohol consumption, positive experience of emotional, physical, and sexual violence during pregnancy, and preference of birth method. Experience of emotional, physical and sexual violence during pregnancy was assessed with three questions (one for each), with three “never”, “sometimes”, and “most often”, options. During analysis, the “sometimes” and “most often” responses were combined and was considered as positive experience of violence.

The Spielberger’s state anxiety inventory was used to measure anxiety state, which is a 20-item scale with total score range of 20–80. Scores 20–40 are regarded as mild anxiety, 41–54 as moderate anxiety, and ≥ 55 as severe anxiety [25]. Fear was assessed using question no.9 “I feel frightened” in the Spielberger scale. Options “not at all” and “somewhat” were considered as low fear, and options “moderately so” and “very much so” as high fear. For adults, it takes about 10 min to complete the full 40-item state-trait anxiety inventory [26]. Considering the study situation, which we had to discontinue the interviews when the participants had uterine contraction or severe pain, we used only state anxiety inventory which took in average about 10 min to be completed.

Dehydration was diagnosed by examining for the following signs and symptoms: dry mouth and lips, thirst, difficult speaking due to a sticky dry feeling in the mouth, and difficult swallowing [27]. Existence of any of the symptoms lasted more than 30 min or any of the signs was regarded as “dehydration”. In case of diagnosis of dehydration, the women were given liquids.

The labour progression checklist was completed during labour, with labour dystocia diagnosed using Zhang’s guideline [28]. Labour interventions, e.g., induction, augmentation, and administration of remifetanil were completed via observation.

Birth satisfaction and views about staff support was assessed 12–24 h post childbirth. The 10-item Iranian (Persian) version of the birth satisfaction scale (IP-BSS, Appendix 2) was used to assess birth satisfaction, which is scored using a five-point Likert scale (0 to 4). Total scores range from 0 to 40, with higher scores representing greater birth satisfaction [29]. The IP-BSS-R has been validated using data from this study and the results were presented in another paper. Original version of the scale was developed in the UK in 2014 [29]. It is now internationally considered the instrument of choice for assessing birth satisfaction [30] and various versions of it have been validated, including the US-BSS-R [31], Greek-BSS-R [32], Turkish-BSS-R [33], Australian-BSS-R [34], Italian-BSS-R [35], and Spanish-BSS-R [36]. The two subscales (satisfaction with doctor and nurse-midwife, items 17–33) of the Mackey childbirth satisfaction rating scale [37] was used to measure staff support. The Mackey scale has been already validated in Iran [38].

Validity of demographic characteristics and labour progression checklist were determined using content validity. Before study commencement, test-retest reliability was evaluated for sensitive socio-demographic data such as domestic violence, sufficiency of household outcome, also for the Mackay and IP-BSS-R scales by asking the related questions twice from 20 women with 14 ± 2 day interval. The first assessment was conducted at the same time-point when data for the main study was collected (socio-demographic data at cervical dilatation of 4–6 cm and the Mackay and IP-BSS-R 12–24 h post childbirth) using face-to-face interview and the re-test data was collected through phone interview. Kappa and intra-class correlation (ICC) coefficients calculated for the qualitative and quantitative variables, respectively, were higher than 0.90 in all of them. Internal consistency was measured for the scales using the Cronbach’s alpha coefficient, which were found to be satisfactory; α = 0.94 for the Spielberger state anxiety inventory, α = 0.97 for nurse and physician sub-scales of the Mackay satisfaction scale, and α = 0.96 for the IP-BSS-R. Also, correlation of IP-BSS-R scores for data collected at the 12–24 h and 40–45 days after birth for the first 210 participants was strong (0.91). As reported in another paper in detail, most other validity measurements were also conducted for the IP-BSS-R, which all confirmed the tool validity to assess birth satisfaction.

### Sample size and statistical analysis

For linear regression equations using six or more predictors, a minimum of 10 participants per predictor variable are required. However, about 30 participants per variable provide better power to detect a small effect size [39]. Therefore, the (*n*=700) sample in this study was sufficient to detect more than 20 possible predictive variables, even variables with small effect size.

Data was analyzed using SPSS-version 21 (SPSS, Chicago, IL, USA). Normal distribution of the birth satisfaction score was confirmed using skewness or kurtosis. The univariate general linear model was used to examine the relationship between each probable predictive variable with the overall birth satisfaction score. To determine pre-labour, during-labour and overall predictors of birth dissatisfaction, after creating dummy variables for qualitative variables with more than two options, all of the variables with *p* < 0.2 in the unadjusted general linear models were entered into multiple linear models with backward strategy. Sidak was used to adjust for the multiple comparisons. Linear regression assumptions such as normality of residuals and no perfect multicollinearity were checked before applying the models. Adjusted R squared was calculated to determine the proportion of the variation in the score explained by the independent variables.

## Results

### Participant characteristics

Data was collected between Oct 2018 and Jun 2019. 26 out of the 700 women were detected as multivariate outliers using Mahalanobis distances [40] and therefore were excluded from analysis. Among the 674 remaining women, 340 (50.4%) were from Taleghani hospital, 382 (57%) were primiparous. 41 (6.1%) had emergency C-section which all was conducted by the residents. Among those with vaginal delivery, majority (86%) were attended by the residents, 11% by midwifery students and 3% by midwives. There was no instrumental childbirth. Labour induction was conducted in 43% of participants and episiotomy in majority of those with vaginal births (95% in primiparous and 67% in multiparous). None of the women reported the use of water pipe or alcohol during their pregnancy and only one woman reported tobacco smoking during pregnancy. Only 5 women were employed. The mean age of the women was 26.1 (SD 6.4) years. Mean score of the total satisfaction score was 23.85 (SD 6.48).

### Association of the pre- and during- labor factors with the birth satisfaction score

In the unadjusted analysis, the pre-labour variables which correlated with low birth satisfaction score included: insufficient household income, being primiparous, no attendance at birth preparation classes, experience of emotional and sexual violence during pregnancy, women’s preference for C-section, and gestational age of 40^0^ weeks or more. The during-labour variables were: dehydration, labour induction and labour augmentation with oxytocin, abnormal fetal cardiogram, receiving remifentanil, labour dystocia, high fear, moderate or severe anxiety state, insufficient support by staff, vaginal birth with episiotomy and/or tear, emergency C-section, and no breast feeding at 1st hour post childbirth. There was no significant differences between women of diverse age, educational levels, with or without planned pregnancy, and with or without experience of physical violence, in relation to satisfaction score (Table 1).

**Table 1:** Birth satisfaction score by pre- and during- labour factors (*n* = 674)

Pre-labour Factors	n	Mean (SD)	Mean difference	During labour factors	n	Mean (SD)	MD (95% CI)
**Hospita**l				**Woman dehydration**^b^			
Taleghani	340	23.8 (6.6)	−0.02 (− 1.0 to 1.0)	No	350	26.4 (5.9)	reference
Al-zahra	334	23.9 (6.3)	reference	Less than 3 h	266	21.8 (6.0)	−4.6 (− 5.7 to − 3.2)^***^
**Age (year)**				More than 3 h	58	18.0 (4.1)	−8.4 (− 10.3 to − 6.4)^***^
≤ 20	116	24.1 (5.8)	0.3 (−1.0 to 1.9)	**Labour Induction with oxytocin**			
21–34	477	23.8 (6.5)	reference	No	384	27.0 (5.6)	reference
35+	81	24.0 (7.1)	0.2 (− 2.0 to 1.7)	Yes	290	19.7 (5.0)	−7.3 (−8.1 to − 6.5)^***^
**Woman education**^a^				**Labour Augmentation with oxytocin**			
No or low	372	24.1 (6.4)	reference	No	196	28.9 (4.7)	reference
High	302	23.6 (6.6)	−0.5 (−1.5 to 0.5)	Yes	478	21.7 (5.9)	−7.2 (−8.2 to − 6.3)^***^
**Views on household Income**				**Amniotomy**			
Sufficient	304	24.7 (6.6)	reference	No	357	24.5 (6.6)	reference
Insufficient	370	23.2 (6.3)	−1.5 (−2.5 to − 0.5)^**^	Yes	317	23.1 (6.2)	−1.4 (− 2.4 to − 0.4)^**^
**Parity**				**Abnormal fetal cardiogram**			
Primiparous	382	23.1 (6.2)	−1.8 (−2.8 to − 0.9)^***^	No	653	24.0 (6.5)	reference
Multiparous	292	24.9 (6.7)	reference	Yes	21	**19.4 (5.5)**	−4.6 (−7.4 to − 1.8)^**^
**Planned pregnancy**				**Receive remifentanil**			
Yes	558	24.1 (6.4)	reference	No	557	24.3 (6.4)	reference
No	116	23.8 (6.5)	0.3 (−1.0 to 1.6)	Yes	117	**21.5 (6.4)**	−2.81 (−4.1 to − 1.5)^***^
**Attendance at birth preparation classes**				**Labour dystocia**			
Yes	70	25.4 (6.2)	reference	No	335	28.8 (4.1)	reference
No	604	23.7 (6.5)	−1.7 (−3.4 to −0.1)^*^	Yes	339	**18.9 (4.2)**	−9.9 (− 10.6 to − 9.3)^***^
**Woman experience of violence during pregnancy**				**Fear**^c^			
**Physical violence**				Low	285	28.7 (4.9)	reference
No	618	24.0 (6.5)	reference	High	389	20.3 (5.1)	−8.4 (−9.2 to − 7.6)^***^
Yes	56	22.4 (6.4)	−1.6 (−3.3 to 0.2)	**Anxiety state**^c^			
**Emotional violence**				Mild	232	30.2 (3.6)	reference
No	484	24.6 (6.3)	reference	Moderate	175	23.9 (4.9)	−6.2 (−7.2 to − 5.3)^***^
Yes	190	21.9 (6.5)	−2.7 (−3.7 to − 1.6)^***^	Severe	267	18.3 (3.7)	−11.8 (−12.7 to − 11.0)^***^
**Sexual violence**				**Support by staff**^b^			
No	600	24.4 (6.4)	reference	Insufficient	109	16.4 (3.4)	−8.8 (−10.0 to − 7.7)^***^
Yes	74	19.4 (5.4)	−5.0 (−6.5 to − 3.5)^***^	Sufficient	565	25.3 (5.9)	reference
**Woman preference of birth**				**Mode of childbirth**			
Vaginal or no matter	578	24.3 (6.4)	reference	Vaginal with no epi/tear	85	27.2 (5.8)	reference
Cesarean	96	21.1 (6.0)	−3.2 (−4.6 to − 1.9)^***^	Vaginal with epi or tear	507	24.2 (6.2)	−2.9 (− 4.8 to − 1.0)^***^
**Gestational age** (weeks)				Vaginal with epi and tear	41	18.9 (5.5)	−8.2 (−11.2 to − 5.2)^***^
37^0^ to 39^6^	427	24.5 (6.4)	reference	Emergency CS	41	16.9 (4.0)	−10.3 (− 13.3 to − 7.3)^***^
40^0^ or more	247	22.7 (6.4)	− 1.77 (−2.8 to − 0.8)^**^	**Breast feeding at 1st h after childbirth**			
				Yes	618	24.2 (6.4)	reference
				No	56	20.3 (5.6)	−4.2 (−5.9 to − 2.4)^***^

*epi* Episiotomy, *CS* Cesarean section, * *P* < 0.05, ** *P* < 0.005, *** *P* < 0.001

All analysis were done using univariate general linear model

Birth satisfaction was assessed by Iranian (Persian) version of the birth satisfaction scale-revised (IP-BSS-R) with range score of 0–40 (the higher score, the higher satisfaction)

^a^15 were illiterate, 357 had low education (153 had elementary, 204 Junior high school/guidance), 240 senior high school and 62 university education, ^b^existence of one of dehydration signs or symptoms (dry mouth and lips, thirst, dizziness, weakness, trouble swallowing dry food, dry, sticky mouth that makes it hard to talk, a swollen, cracked or dry tongue) [27] . ^c^Anxiety was assessed using the Spielberger’s State Anxiety Inventory (scores 20–40: mild, 41–54: moderate, ≥ 55: severe anxiety). Fear was assessed using one question “I feel frightened” with four options (not at all/somewhat: low fear, moderately so/very much so: high fear [25]. Staff support was assessed using nurse and physician subscale of Mackey satisfaction tool (score range:17–85, scores 51 or less: insufficient, 52 or more: good support [37]

### Pre- labour predictors of birth satisfaction score

The predictors included being primiparous (β = − 0.215), sexual (β = − 0.179) and emotional (β = − 0.105) violence during pregnancy, gestational age more than 40 weeks (β = − 0.138), woman preference for caesarean section (β = − 112), no attendance at birth preparation classes (β = − 0.096), insufficient household income (β = − 0.084). The proportion of variation in the satisfaction score explained by these independent variables was 14% (Table 2).

**Table 2:** Pre-labour and during labour factors predictors of dissatisfaction of childbirth (*n* = 674)

Predictors	Beta	B (95% CI)	P	Beta	B (95% CI)	P
1. Pre-labour predictors^*^						
Constant	–	29.7 (27.9 to 31.5)	< 0.001	–	–	–
Primiparous	− 0.215	− 2.8 (− 3.8 to − 1.8)	< 0.001	–	–	–
Sexual violence during pregnancy	− 0.179	− 3.7 (− 5.4 to − 2.0)	< 0.001	–	–	–
Gestational age of 40^0^–41^6^ w (Ref: 37^0^–39^6^)	− 0.138	− 1.9 (− 2.8 to − 0.9)	< 0.001	–	–	–
Woman preference for Cesarean section	− 0.112	− 2.1 (− 3.6 to − 0.5)	0.003	–	–	–
Emotional violence during pregnancy	− 0.105	− 1.5 (− 2.7 to − 0.3)	0.012	–	–	–
No attendance at birth preparation classes	− 0.096	− 2.0 (− 3.6 to − 0.5)	0.009	–	–	–
Perceived insufficient household income	− 0.084	− 1.1 (− 2.1 to − 0.1)	0.025	–	–	–
**2. During-labour predictors**†	Excluded woman fear			Excluded woman anxiety state		
Constant	–	31.4 (30.7 to 32.2)	< 0.001	–	31.6 (30.7 to 32.4)	< 0.001
Sever anxiety state (Ref: mild)	−0.455	− 6.0 (− 6.9 to − 5. 2)	< 0.001	–	–	–
Labour dystocia	− 0.301	− 3.9 (− 4.7 to − 3.1)	< 0.001	− 0.382	− 4.9 (− 5.7 to − 4.1)	< 0.001
High fear	–	–	–	− 0.239	− 3.1 (− 3.8 to − 2.5)	< 0.001
Moderate anxiety state (Ref: mild)	−0.236	−3.5 (− 4.2 to − 2.8)	< 0.001	–	–	–
Insufficient support by staff	−0.139	−2.7 (− 3.4 to − 1.9)	< 0.001	−0.177	−3.1 (− 3.91 to − 2.3)	< 0.001
Mode of Birth (Ref: vaginal with no episiotomy/no tear)						
Vaginal birth with episiotomy and tear	− 0.139	−3.8 (− 4.9 to − 2.5)	< 0.001	−0.158	−4.3 (− 5.6 to − 3.0)	< 0.001
Emergency cesarean section	− 0.113	− 2.9 (− 4.2 to − 1.7)	< 0.001	− 0.134	− 3.5 (− 4.8 to − 2.2)	0.001
Vaginal birth with episiotomy or tear	− 0.055	− 0.8 (− 1.6 to − 0.1)	0.034	− 0.064	− 1.0 (− 1.8 to − 0.1)	0.021
Labour induction with oxytocin	−0.098	− 1.3)- 1.9 to − 0.7)	< 0.001	−0.103	−1.3)- 2.0 to − 0.7)	< 0.001
Labour augmentation with oxytocin	–	–	–	− 0.076	−1.1 (− 1.8 to − 0.3)	0.004
Woman dehydration (Ref: no dehydration)						
Dehydration + 3 h	− 0.058	−1.3 (− 2.3 to − 0.3)	0.008	−0.073	−1.7 (− 2.7 to − 0.7)	0.002
Dehydration < 3 h	−0.045	− 0.6 (− 1.2 to − 0.03)	0.039	−0.045	− 0.6 (− 1.2 to 0.01)	0.053
**3. Overall predictors**^**‡**^	Excluded woman fear			Excluded woman anxiety state		
Constant	–	31.4 (30.8 to 31.8)	< 0.001	–	31.3 (30.8 to 31.9)	< 0.001
Severe anxiety state (Ref: mild)	−0.426	−5.6 (− 6.5 to − 4.8)	< 0.001	–	–	–
Labour dystocia	−0.324	−4.2 (− 5.0 to − 3.4)	< 0.001	−0.414	−5.4 (− 6.15 to − 4.6)	< 0.001
Moderate anxiety state (Ref: mild)	−0.222	−3.3 (− 4.0 to − 2.6)	< 0.001	–	–	–
High fear	–	–	–	− 0.239	− 2.8 (− 3.4- to − 2.1)	< 0.001
Insufficient support by staff	−0.157	− 2.7 (− 3.5 to − 2.0)	< 0.001	−0.183	− 3.2 (− 3.10 to − 2.4)	< 0.001
Mode of Birth (Ref: vaginal with no episiotomy/no tear)						
Vaginal birth with episiotomy and tear	− 0.109	− 2.9 (− 3.9 to − 1.9)	< 0.001	− 0.123	− 3.3 (− 4.4 to − 2.3)	< 0.001
Emergency caesarean section	−0.076	−2.0 (− 3.0 to − 1.0)	< 0.001	−0.088	−2.3 (− 3.3 to − 1.2)	< 0.001
Primiparous	− 0.093	−1.2 (− 1.7 to − 0.7)	< 0.001	− 0.105	−1.4 (− 1.9 to − 0.8)	< 0.001
Labour induction	− 0.088	−1.1)- 1.7 to − 0.5)	< 0.001	− 0.098	−1.3)- 2.0 to − 0.6)	< 0.001
Labour augmentation	–	–	–	− 0.079	−1.1 (− 1.8 to − 0.4)	0.002
Sexual violence during pregnancy	−0.060	−1.2 (− 2.1 to − 0.4)	0.003	−0.062	−1.3 (− 2.2 to − 0.4)	0.004
Woman dehydration + 3 h (Ref: no dehydration)	−0.047	−1.1 (− 2.1 to − 0.1)	0.029	−0.049	−1.1 (− 2.1 to − 0.2)	0.022
Woman dehydration < 3 h (Ref: no dehydration)	−0.036	− 0.5 (− 1.1 to 0.07)	0.099	–	–	–

Birth satisfaction was assessed by Iranian (Persian) version of the birth satisfaction scale-revised (IP-BSS-R) with range score of 0–40 (the higher score, the higher satisfaction)

All analysis were done using multiple linear regression model with backward strategy. Sidak was used to adjust for the multiple comparisons

* Adjusted for all other pre-labour variables with a relation of *p* < 0.2 in the univariate analysis (variables of attendance at pregnancy classes, parity, history of abortion, interval between previous childbirth, household income, planned pregnancy and physical violence were removed from the model). adjusted *R*^*2*^ = 0.137

† Adjusted for all during labour variables with a relation of *p* < 0.2 in the univariate analysis (variables of woman dehydration, labour augmentation, amniotomy, amnion sac status, fetal cardiogram condition, administeration of remifentanil and time of breastfeeding were removed from the model. Adjusted *R*^*2*^ = 0.749 when excluded fear and adjusted *R*^*2*^ = 0.717 when excluded woman anxiety variable

‡ Adjusted for all variables entered in the above models. Adjusted *R*^*2*^ = 0.756 when excluded fear and adjusted *R*^*2*^ = 0.725 when excluded woman anxiety variable

### During-labour predictors of low birth satisfaction score

There was collinearity between fear and anxiety factors. Therefore, we conducted two models: 1) 1) with excluding factor of woman fear, 2) with excluding factor of woman anxiety.

Excluding factor of woman fear, the predictors included severe (β = − 0.455) and moderate (β = − 0.236) anxiety state, labour dystocia (β = − 0.391), insufficient support by staff (β = − 139), vaginal birth with episiotomy and tear (β = − 0.139), emergency C-section (β = − 0.113), labour induction with oxytocin (β = − 0.098), vaginal birth with episiotomy or tear (β = − 0.055), and women dehydrated for more than 3 h (β = − 0.058) and less than 3 h (β = − 0.045). The proportion of variation in satisfaction scores explained by these independent variables was 75% (Table 2).

Excluding factor of woman anxiety, the predictors included the above ones (with small changes in their β) in addition to high fear (β = − 0.239), and labour augmentation with oxytocin (β = − 0.076). The proportion of variation in satisfaction scores explained by these independent variables was 72% (Table 2).

### Overall predictors of low birth satisfaction score

Excluding factor of woman fear, the predictors included severe (β = − 0.426) and moderate (β = − 0.222) anxiety state, labour dystocia (β = − 0.324), insufficient support by staff (β = − 0.157), vaginal birth with episiotomy and tear (β = − 0.109), emergency C-section (β = − 0.076), being primiparous (β = − 0.093), labour induction with oxytocin (β = − 0.088), sexual violence (β = − 0.060), and women dehydrated for more than 3 h (β = − 0.047) and less than 3 h (β = − 0.036). The proportion of variation in satisfaction score explained by all these factors was 76% (Table 2).

Excluding factor of woman anxiety, predictors included the above ones in addition to high fear (β = − 0.239), and labour augmentation with oxytocin (β = − 0.079). The proportion of the variation in satisfaction score explained by all these factors was 72% (Table 2).

## Discussion

The quite high proportion (75%) of variation in satisfaction scores are explained by the during-labour variables, which include severe and moderate anxiety state, labour dystocia, insufficient support by staff, vaginal birth with episiotomy and tear, emergency C-section, labour induction and augmentation with oxytocin, and dehydration. Replacing the factor of anxiety state (assessed by 20 items) with fear (assessed by one item) does not change considerably the proportion. Pre-labour predictors include being primiparous and experiencing sexual and/or emotional violence during pregnancy, gestational age of 40^0^–41^6^ weeks, woman preference for C-section, no attendance at birth preparation classes, and insufficient household income explain only 14% of the variation in satisfaction score. Adding the pre-labour predictors to the during-labour predictors does not increase the explanation proportion.

In overall, results of the current study are in agreement with the literature review, which indicate that supporting women during birth, minimal interventions, and birth preparedness are successful strategies for creating positive perceptions of childbirth [41]. A systematic review also shows that the during-labour factors such as the amount of support from caregivers and the quality of the caregiver-client relationship are more important factors influencing on birth satisfaction compared to some pre-labour factors such as age, socioeconomic status and childbirth preparation [42].

Using the BSS-R scale, mean birth satisfaction score in this study (23.8, SD 6.5) was lower than that in most studies in other countries such as US (32.4, SD 6.4) [31], Australia (30.4, SD 5.9) [34], UK (28.4, SD 5.6) [29], Spain (28.1, SD 6.2) [36], and Greece (27.4, SD 6.2) [32] but was higher than that in Turkey (20.4, SD 6.0) [33]. The high medicalized birth management in Iran may be an important reason for the lower birth satisfaction in the study setting compared to many of the other studies.

Although, WHO recommends that routine or liberal use of episiotomy should not be used for women undergoing spontaneous vaginal birth [5], it was routinely used in the study setting. But in most of above-mentioned countries it is used selectively. For example, its reported rate in a national survey in the US was 25% in 2004 [43] and in a recent study in two largest maternity public hospitals in Greece was 17% [44]. Similar with Iran, episiotomy rate in Turkey is also high (93% in primiparous and 30% in multiparous women) [45]. In the current study, both episiotomy with or without a tear was associated with low satisfaction. Third degree perineal tear was also identified as an important predictive factor in a study in Canada [46]. Higher pain and discomfort following episiotomy especially extended one may affect women’s perceptions of disturbances to the structure of their reproductive organs and perceived potential for future enjoyment of sex [47].

In tertiary level teaching hospitals in Iran, most deliveries are attended by obstetrics residents. In our study, only 14% of births were attended by midwives or midwifery students. Studies show that birth attendance by skilled motivated midwives could improve more than 50 maternal and neonatal outcomes including reduced anxiety during labour and satisfaction with childbirth experience [48]. According to the Cochran review, women who received midwife-led continuity models of care were less likely to experience intervention and more likely to be satisfied with their care than those who received other models of care [49]. Therefore, WHO emphasizes on providing care based on the midwife-led models for promoting birth satisfaction [5].

In this study, severe anxiety state was the strongest predictor, and moderate anxiety was a strong predictor of low satisfaction. A possible explanation is the relationship of high anxiety with feeling of no or low personal control during labour and birth [50] and relationships of low personal control with maternal birth dissatisfaction [37, 51]. Another explanation could be the positive relationship of high anxiety state with more severe labour pain [52], and relationships of experiencing pain with dissatisfaction [37].

The result of high fear as a strong predictor of low satisfaction, could be considered consistent with the study conducted with Swedish and Australian women [53], which indicate that fearful women experience psychological distress significantly increases chances of having a negative childbirth experience. Other studies also indicate high fear of birth as predictive factor of negative birth experience [54–56]. Childbirth satisfaction and childbirth experience are highly correlated [57].

In our study, labour dystocia was a strong predictor of low satisfaction, which is in keeping with other study results [46, 58]. Prolonged birth was identified as a predictive factor of birth satisfaction in Canada [46] and as an associated factor in a Swedish study [58]. Administration of medical interventions and having severe labour pain which are associated with labour dystocia may have been one cause of low satisfaction.

Similar results about insufficient support from midwife/nurse and/or physician, as a strong predictor of low childbirth satisfaction, is also reported in other studies [10, 11]. Also, in a systematic review it was indicated that perceived unhelpfulness of care providers is a predictor of childbirth dissatisfaction [42].

In a Swedish study, emergency C-section was the strongest predictor of childbirth dissatisfaction [59]. Yet in our study it was an important predictor, but not the strongest one, with its effect weight less than vaginal delivery with episiotomy and tear (extended episiotomy). This may have been related to Iranian women having a higher preference for C-section compared to Swedish women. In Sweden, 7% of pregnant women reported a preference for having a C-section [60]. But in the present study which those with planned C-section were not included, it was 14%. In another Iranian study, the preference was 32% [61]. C-section rate in Sweden is 17.4% while in Iran is 45.6% [62]. High education, fear of vaginal birth and doctor’s recommendation have been mentioned as the most non-obstetric factors influencing on the high C-section rate in the country [63].

Our results are also consistent with previous studies which show associations between labour induction and augmentation with low satisfaction [59] and negative birth experience [58, 64]. Increased severity of pain from interventions may explain these associations.

In previous studies, obstetric interventions, including emergency C-section [59, 65] and labor induction [59] were significantly related to dissatisfaction with childbirth and negative birth experience. Other factors, such as lack of control, insufficient/no involvement in decision-making, and complications for mother or child may also explain the associations.

Being primiparous was a strong pre-labour predictor of low satisfaction, with its predictive weight reduced considerably in the overall model which included all pre and during-labour factors. Higher anxiety, fear of birth among women with no experience of birth could explain some of this association. Also, only one tenth of participants in the current study attended birth preparation classes. Being primiparous was associated with low birth satisfaction in a US study [65], and Swedish study [58], with a review supporting low birth satisfaction amongst primiparous women [42].

In current study, reported experience of sexual abuse during pregnancy was a strong pre-labour predictive factor of the low satisfaction, which was also included in the overall model. Its weigh was reduced in the overall model in both pre- and during-labour variables (change in beta from − 0.179 to − 0.071). This association may be related to high fear of birth and anxiety among such women. A Norwegian study showed history of abuse was common among women with high fear of birth compared to those with moderate fear (76% vs 39%) [66]. Although emotional abuse was associated with low satisfaction, its weight was low in the pre-labour model, and it was not included in the overall model. Studies in Norway [55] and Canada [67] also indicate that a history of abuse is an associated factor with negative birth experience, with type of abuse not reported in these studies.

Limited knowledge exists about the association of dehydration during labour and birth satisfaction. Although in the unadjusted analysis, the mean difference of satisfaction score between women with > 3 hour compared with those with no dehydration was high (β = -8.4). In the adjusted analysis, this factor gained lowest weight amongst all of the predictive factors. Possible reason for this change may be its multicollinearity with other factors. Dehydration may result in some metabolic disorders, which in turn could result in the dysfunction of uterine muscles and prolonged labour [68]. Another possible explanation could be women’s perceptions of lack of staff attention to their personal needs.

No other study reported associations between gestational age of 40^0^–41^6^ weeks and birth satisfaction. In the current study, this was a pre-labour predictor of low birth satisfaction, yet was removed from the overall model. In the setting of this study, women at gestational age of 40 weeks or more, even with no labour pain, were hospitalized. Long duration of stay in hospital and interventions, such as induction and vaginal examinations, may have resulted in fatigue and increased concern and anxiety, which in turn lowers satisfaction.

Our reports of higher birth satisfaction in those who attended birth preparation classes, is also in line with results of other studies in Iran [69] and other countries [70–72]. In a Canadian study, degree of awareness of events during birth was the strongest predictor of women’s perceptions [56]. Possible explanations for these relationships, are that education and exercise classes could decrease fear and anxiety [73], increase confidence in ability to cope [70], result in shorter duration of labour [71], and increase ability to push spontaneously [74].

Our associations of insufficient household income and low satisfaction are in keeping with other studies [55, 75], with concerns about being able to pay for care and a new infant augmenting reduced satisfaction.

### Strengths and limitations

A strength of this study is that it is one of the first to investigate predictors of birth satisfaction in Iran. Also, the relatively large sample size provided sufficient power to investigate the high number of factors associated with low birth satisfaction, even those factors with a small effect size. Assessing the possible predictive factors before assessment of satisfaction may also have reduced information bias.

Assessing some of the possible risk factors at early stage of active phase of labour might affect the women’s ability to participate in an interview and respond correctly to the questions. However, the high skills of the investigator who was an experienced midwife for effective communication with clients prevent the problem. Results of the validation tests such as ICC and Cronbach’s alpha also confirmed validity of the collected data.

Assessing birth satisfaction at 12-24 hours post birth and before discharge from hospital could be considered a limitation of this study. However, as reported in the paper, there was a strong correlation (*r* = 0.91) between satisfaction scores assessed at 12-24 hour and at 40-45 days post birth.

Having no information about women’s psychological distress, e.g., anxiety before pregnancy and labour, could be a further limitation of this study. Consequently, we cannot judge associations between anxiety during pregnancy and birth satisfaction.

This study was conducted at tertiary teaching settings with many complicated cases referred from other centers where obstetrics residents were the main caregivers during labour and birth. Therefore, the results may not be generalizable to the primary or secondary level settings where midwives are main responsible person for the childbirths and mostly provide services for low risk women or may not be generalizable to the private settings where midwives and obstetricians provide services for the women during labour and birth.

Due to the nature of observational studies, we also cannot consider cause of effects, with further studies to address controllable factors recommended.

## Conclusions

Based on this study results, the during-labour predictors explain most of the variance in childbearing women’s birth satisfaction scores. Most predictors of the low childbirth satisfaction, e.g., anxiety, insufficient support from staff during labour, maternal dehydration, and allied interventions are controllable. Appropriate consultation with couple during pregnancy and encouraging to attend birth preparation classes may also reduce some factors such as violence, fear and anxiety.

Hence, responding to women’s physical and psychological needs, reducing interventions, meeting women’s desire for information during pregnancy and childbirth, and women’s involvement in decision making which are in line with woman-centred models of childbirth care, in particular through midwife-led models, could enhance the birth satisfaction.

## Supplementary information

**Additional file 1.**

**Additional file 2.**

## Data Availability

The datasets used and analysed during the current study are available from the corresponding author on reasonable request.

## Abbreviations

WHO: World health organization
CS: Caesarean section
IP-BSS-R: Iranian (Persian) birth satisfaction scale-revised
CI: Confidence interval
