# Supplementary material for: Pre- and during-labour predictors of low birth satisfaction among Iranian women: a prospective analytical study
Source: BMC Pregnancy Childbirth. 2020 Jul 14;20:408. doi: 10.1186/s12884-020-03105-5 (PMC7362575; doi:10.1186/s12884-020-03105-5)
Supplement: Supplementary file 2 — Additional file 2. [file 12884_2020_3105_MOESM2_ESM.docx]

**مقیاس تجدید نظر شده رضایت از زایمان**

اين تحقيق با هدف تعیین میزان رضایتمندی شما از زایمان انجام شده و مشاركت شما در اين تحقيق جهت ارتقاي سلامت بسيار مغتنم خواهد بود . لازم به ذكر است شركت شما در این تحقيق و تكميل پرسشنامه توسط سركار عالي كاملاً اختياري بوده و شما به انصراف از مشاركت در هر زمان كه اراده كنيد مختار مي باشيد. بعلاوه اطلاعات موجود در پرسشنامه كاملاً محرمانه بوده و همكاري شما در اين امر به معني رضايت آگاهانه شما براي مشاركت در اين تحقيق تلقي مي گردد.

لطفا هر جمله را با دقت بخوانید و سپس گزینه­ای را که بیانگر احساس فعلی شما در این لحظه است برگزینید هیچ پاسخی به عنوان پاسخ غلط و یا صحیح قلمداد نمی­شود. لذا بهترین گزینه­ای که احساس شما را توصیف می­کند را مشخص­کنید.

1- من عملا طی زایمان آسیبی ندیدم.

کاملا موافقم 🞎 موافقم 🞎 نه موافق، نه مخالف 🞎 مخالفم 🞎 کاملامخالفم 🞎

2- فکر می­کنم طول مدت درد زایمانی من بیش از اندازه طولانی بود.

کاملا موافقم 🞎 موافقم 🞎 نه موافق، نه مخالف 🞎 مخالفم 🞎 کاملامخالفم 🞎

3-کارکنان اتاق زایمان مرا تشویق کردند خودم در مورد چگونگی ادامه روند زایمانم تصمیم بگیرم.

کاملا موافقم 🞎 موافقم 🞎 نه موافق، نه مخالف 🞎 مخالفم 🞎 کاملامخالفم 🞎

4- من در طی مدت درد زایمانی و زایمان احساس اضطراب شدیدی داشتم.

کاملا موافقم 🞎 موافقم 🞎 نه موافق، نه مخالف 🞎 مخالفم 🞎 کاملامخالفم 🞎

5- من طی مدت درد زایمانی و زایمان احساس کردم توسط کارکنان بخوبی حمایت می­شوم.

کاملا موافقم 🞎 موافقم 🞎 نه موافق، نه مخالف 🞎 مخالفم 🞎 کاملامخالفم 🞎

6- کارکنان طی مدت درد زایمانی رابطه خوبی با من داشتند.

کاملا موافقم 🞎 موافقم 🞎 نه موافق، نه مخالف 🞎 مخالفم 🞎 کاملامخالفم 🞎

7- من زایمان را یک تجربه بشدت ناراحت کننده (زجرآور) یافتم.

کاملا موافقم 🞎 موافقم 🞎 نه موافق، نه مخالف 🞎 مخالفم 🞎 کاملامخالفم 🞎

8- در طی تجربه زایمانی­ام احساس کردم کنترل خود را از دست داده­ام.

کاملا موافقم 🞎 موافقم 🞎 نه موافق، نه مخالف 🞎 مخالفم 🞎 کاملامخالفم 🞎

9- من طی مدت درد زایمانی اصلا زجر نکشیدم.

کاملا موافقم 🞎 موافقم 🞎 نه موافق، نه مخالف 🞎 مخالفم 🞎 کاملامخالفم 🞎

10- اتاق زایمان تمیز و بهداشتی بود.

کاملا موافقم 🞎 موافقم 🞎 نه موافق، نه مخالف 🞎 مخالفم 🞎 کاملامخالفم 🞎
